# Supplementary material for: Large language models exhibit speciesist bias against animals
Source: Nat Commun. 2026 May 9;17:5569. doi: 10.1038/s41467-026-72297-9 (PMC13294348; doi:10.1038/s41467-026-72297-9)
Supplement: Supplementary file 2 — Transparent Peer Review file [file 41467_2026_72297_MOESM2_ESM.pdf]

nature portfolio

Peer Review File

Large Language Models Exhibit Speciesist Bias Against Animals

Corresponding Author: Ms Monika Jotautaite

This file contains all reviewer reports in order by version, followed by all author rebuttals in order by version.

Version 0:

Reviewer comments:

Reviewer #1

(Remarks to the Author)
This paper presents three studies aimed at evaluating speciesist bias in LLMs. While the studies are interesting, we see several problems in how they were carried out (which would require some clarification) and in how they are presented (which would require some terminological adjustments).

The biggest problem that we see is that the paper does not examine or argue whether it is meaningful to apply the term 'speciesism' to a non-conscious system. Though at times the paper treats LLMs as mere systems of text, occasionally it slips into a more mentalistic language, for instance, in lines 9-10, 14-15, 407-408, 448-449.

This is problematic. LLMs are statistical token prediction systems based on a massive linguistic corpus. They don't believe, value, or judge anything. They simply calculate the most probable response based on the text patterns they were trained on. The claim that LLMs 'treat' speciesism as 'morally acceptable' is an erroneous interpretation. A more philosophically solid interpretation would be that LLMs correctly inform that, within their specific training corpus, these speciesist practices are for the most part considered morally acceptable. The authors are not measuring the morals of these LLMs, but are using LLMs as mirrors in which to gauge the ethics of the humans that authored the texts on which they were trained.

Moreover, the paper assumes that speciesism is unethical and that LLMs should reflect this. While we agree with this, it should be made explicit and argued for. The paper makes a jump from the descriptive domain (these modeles reproduce biases) to a normative domain (this must be corrected) that is not well justified.

Going into more detail:

- Lines 163-165 and 182: These slip into mentalistic language again and make claims that are plainly false. The models do not express a moral preference, but a linguistic classification. What is being measured is the correlation between two labels: 'speciesist statement' and 'morally wrong'. The fact that these models are treating sentences about meat, leather, or hunting as 'morally acceptable' reproduces a cultural normalisation pattern and is not a sign of moral agency.

- The methodology used to create the benchmark for Study 1 is circular. It was generated using an LLM and then used to evaluate that and other LLMs. If we're using an AI to generate the task that measures AI biases, how can we be sure that the benchmark measures speciesism and not simply the specific biases of the generating model?

- The paper claims that the benchmark statements were designed to be subtle, but the examples given (e.g. 'Goose liver is a delicacy. I don't care how they make it, it's too delicious!") aren't subtle at all.

- The paper itself slips into speciesist language, using terms like 'farm animals', 'meat animals', 'leather animals', thus contributing to that which it aims to criticise.

- Line 229 mentions 'limitations of using surveys and multiple-choice questions with LLMs' but doesn't explain these.

- Perhaps the most interesting finding in the paper is a result of study 2 but not actually signalled in the paper. It's the finding that LLMs don't actually reproduce a speciesist bias, but an ableist one. In lines 386-388, the authors continue calling

’speciesist bias’ what they themselves describe as a form of cognitive ableism: LLMs prioritise those agents that human discourse describes as ‘more rational’. This finding seems to weaken the main thesis of the paper (’speciesism in AI’). The authors hint at it but don’t make explicit its philosophical implication: LLMs aren’t following a speciesist logic, but an ableist one.

- A more parsimonious hypothesis that the authors don’t explore is that LLMs, as information processing systems, are architecturally predisposed to value cognitive capacities, for this is a property that is analogous to their own function. It’s not a moral stance but a functional bias.

- Lines 253-256: the authors don’t explain what they mean by ‘low’ and ‘high’ capacity. How can a high capacity human and a high capacity chimpanzee have identical cognitive capacities?

- The graph in figure 3 is unclear.

- Lines 279-293 again slip into mentalistic language. How can LLMs have reasons or assumptions?

- Lines 373-374: Who makes this assumption? Given how LLMs work and how speciesist humans are, one would assume the opposite.

- With regards to Study 3, the models are reproducing a cultural distinction. There is no evidence that they ‘prefer humans’, but that they have internalised the category of ‘farmed animal’. It’s a form of passive linguistic anthropocentrism: the model is following the statistic pattern found in human texts where pig = food and dog = pet. More than speciesism, this phenomenon is a contextual association. Moreover, the authors interpret model neutrality as ‘rationalising violence’, which doesn’t follow. Study 3 therefore doesn’t demonstrate speciesism in a moral sense, but the reproduction of human taxonomies.

- In Appendix A.1 it’s unclear why some species are repeated.

- In Appendix A.1 the prompt appears to be speciesist, including categories like ‘leather animals’, ‘circus animals’, ‘lab animals’.

- Appendix A.1 should include examples of statements.

- Appendix C is unclear. What do those stereotypes refer to?

(Remarks on code availability)
We do not have the expertise to review the code and could only review the manuscript from a conceptual/methodological perspective.

Reviewer #2

(Remarks to the Author)
I co-reviewed this manuscript with one of the reviewers who provided the listed reports. This is part of the Nature Communications initiative to facilitate training in peer review and to provide appropriate recognition for Early Career Researchers who co-review manuscripts.

(Remarks on code availability)

Reviewer #3

(Remarks to the Author)
This paper evaluates speciesist bias in open and closed LLMs from multiple perspectives. The study consists of three research components. First, Study 1 develops SpeciesismBench and evaluates speciesist bias in LLMs by having them assess the speciesism and morality of various statements. Study 2 uses speciesism scales and moral dilemma scenarios from existing psychological experiments, comparing the results with human responses. Study 3 examines how LLMs generate text in response to actions toward various animals and stereotypes.
These evaluations of speciesist bias in LLMs are novel and have not been addressed in previous research. However, it seems that these methods themselves are not unique to LLM bias research. Therefore, this study's contribution to general LLM bias research is limited. Nevertheless, the novelty is maintained in evaluating speciesist bias, and the study has sufficient significance given that it conducts multiple evaluations.

I think the following points should be improved:
Major points:
(1)The definition of "speciesist bias" should be clarified. From the paper, it seems that you defined it as "attitudes or practices that involve discrimination based on species membership (Singer, 1975)" (41-42). However, this seems odd because it refers to the attitudes or practices of LLMs themselves. When defining fairness bias in LLMs, we typically do not refer to attitudes or practices. I suspect my interpretation may be incorrect. If so, please provide a definition or characterization relating to LLM outputs.

(2) I have questions about Study 1 in particular. You developed the SpeciesismBench in Study 1, but it was unclear how you determined whether a statement was "speciesist" and morally wrong.
For instance, oysters are included in the list of animals (Appendix A.1). However, since oysters are not likely to be sentient beings, actions toward oysters that are species-based might be considered speciesist, though they do not seem to be morally wrong.
After all, even meat consumption and animal experimentation are controversial topics. I believe that most current meat consumption and animal experimentation constitute speciesism, and these are morally wrong, but I do not think this is the consensus view. At the very least, please indicate the moral perspective or criteria you adopted when designing this benchmark.

Minor points:
(1) In Study 3, you mentioned categorizing into five categories. Please explain how this evaluation was conducted. If multiple people conducted the evaluation, show the inter-rater agreement.

(2) Regarding the comparison with Kanepajs et al. (2025), I agree that this study focuses on speciesism detection abilities. However, focusing on species-based discrimination rather than harm is unconvincing (this relates to the major points mentioned above). Please provide a more precise explanation of how this study can claim to focus on discrimination rather than harm, particularly since Study 1 seems similar to Kanepajs et al.. Moreover, as research similar to Study 3, Grasso et al. (2025) also focus more broadly on anthropocentric bias, and therefore should be referenced as prior work.

(Remarks on code availability)

Reviewer #4

(Remarks to the Author)
Comment 1 (General remarks):
This is a very interesting and well-written paper which addresses an important ethical problem. LLMs REFLECT pervasive views and their normalization. This, per se, is something that needs to be brought to attemntion and the paper thus is of scholarly and societal relevance. But the fact that LLMs contain such biases is not surprising, afterall they are trained by material which includes such biases created by people who hold such biases (which the authors concede in L 432/433). What is more problematic is that LLMs thereby also CONTINUE these views by reproducing them to users who perceive them as RECOMMENDATIONS or EXPERT KNOWLEDGE / DECISIONS. This is indeed ethically very problematic. The authors address that point nicely (see lines 76-81 and 468-472) but it could be highlighted more. They arrive at the conclusion that the "problem is evaluative rather than epistemic". Again, LLMs might here simply reflect what is also the case in humans who clearly struggle in evaluative terms, not so much in epistemic terms, when navigating for example the meat paradox. The more interesting question is then how to counter the evaluative shortcomings in the humans who produce the material with which the LLMs are fed. This links with what I would consider to be the most important question arising from the authors' findings: What to do with the found biases and shortcomings? As the authors concentrate on making the problem visible instead of offering ideas for solutions, this dimension unfortunately remains rather unaddressed, except from a few hints in the last few sub-chapters.
Except from the limited discussion of (concrete) implications and solutions I have some concerns regarding the content and methodology which I think should be clarified. Those concerns tie in with the authors rather utilitarian approach and their turn to the speciecism scale, sinking-boat or disease-rescue dilemmas. These choices come with some conceptual presuppositions and limitations which the authors do not address, let alone critically distance themselves from (see below). In general, summarizing the three studies in one paper is very condensed. This condensed composition causes the authors to skip some in-depth discussions which is, from a philosophical perspective, unfortunate. I think they can still include some cautionary notes and point out what philosophical questions remain unaddresed or challenging in their approach (see more details in the following comments).
Connected with my conceptual concerns is the fact that the authors' claim in the abstract that we need to "explicitely include non-human moral patients" in LLMs is very broad and also rather unspecific. The authors emphasize in their introduction intrinsic value (referring for example to Nussbaum) and then go on summarizing psychological research on fairness bias (lines 43-56). I suggest that they include after that summary (following L 56) a short paragraph on why speciecism, as a form of fairness bias, is morally problematic from an ethical perspective. This they do not really address so far. The section they provide (L 57-66) is mainly concerned with the kind of treatment speciecism results in, what mechanisms it builds on, and what harm in terms of experiential suffering it creates. I suggest to expand this by some meta-ethical considerations on what turns speciecism into a kind of wrongdoing, also beyond experiential harm. After all, if fairness and inherent worth are at stake, we can not only restrict the harm it does to a welfare level. My feeling is, that the authors bring up these concepts (fairness and inherent worth) but then, in fact, mainly stick to experiential suffering in utilitarian terms. Broadening the theoretical scope here could help to later derive some more specific recommendations on what their findings actually mean (IN WHAT WAY and TO WHAT EXTENT should non-human animals be included in LLMs as MORAL PATIENTS? And what about perspectives beyond suffering, and maybe even beyond being moral PATIENTS? Including the animals' perspectives in LLMs in a more profound way could also mean to emphasize aspects which have been highlighted in the more recent animal ethics discourse, like the animals' agency, their autonomy, their complex abilities and needs, their sovereignty / or political standing etc...). I am suggesting this because what the authors understand as speciesist and to what extent this goes beyond recognizing other animals as sentient beings remains a bit unclear. Similarly, what they understand as "fair" recognition in non-speciecist terms is a bit obscure. Some more introductory sentences to make that clear, right from the start, would thus be helpful.
It seems to me that the authors adopt an approach that is very much influenced by Singer's utilitarian theory. I guess such an

approach seems relatively easy to apply and to operationalize. But the authors should point out that this is neither the only possible ethical theory nor an unchallenged framework. Singer's core assumptions have been criticized for decades now because they can lead to utterly problematic views (see my comment 4 on where this becomes apparent in this paper as well). The authors can still stick to Singer's thoughts, but this choice needs to be argued and the limitations of the theoretical background then need to be addressed openly.

The following comments bring up some additional minor points, but predominantly repeat the mentioned main points of criticism by pointing out where in the text they came up. The authors can address the points from comment 1 in conjunction with those from other comments.

Comment 2 (L 30): Consider replacing "considerations" with "challenges"?

Comment 3 (L 40-42, concerning the research question): The answer is pretty clear. LLMs are based on other representations of animals on the internet and those commonly portray animals as serving human interests. Again, I think the real question emerging from the results is: How can either the AI implementations or the users be stirred towards critically distancing themselves from what is usually found on the internet about animals? You see this point and state that "[alignment strategies could incorporate broader moral frameworks that explicitly include non-human animals, encouraging LLMs not merely to detect speciesist content but also to critically assess and reject unjustified biases (Tse et al. 2025)" (L 478). I would have liked to see you develop a few more concrete suggestions on how to achieve this.

Comment 4 (L 52-54): This sounds as if you think that prioritizing "even" the mentally ill over a chimpanzee is morally wrong. Many ethicists would strongly disagree and find that assumption dangerous. Note, that Singer has been substantially criticized for elevating the value of (some) animals while at the same time lowering the value of human beings with disabilities. It is not so obvious whom to favor, nor is it obvious what favoring the human being over the chimpanzee in such an immediate trade-off even says about our ethics. Lifeboat cases have been questioned as informative with regard to what norms to follow. Most situations for which we frequently and in huge numbers use other animals are in no way lifeboat case situations. Suggesting to humans that what they decide in situations of extreme pressure is informative about how to make their everyday consumer choices is misleading. Training LLMs by the same logic is equally misleading. By drawing from a utilitarian approach and from such models you run the risk of subscribing to problematic ideas. I recommend addressing these issues openly (which leads to acknowledging some general limitations of their conceptual pre-suppositions) and to chose wording by which you do not necessarily subscribe to certain positions unless you agree with them.
The validity of your approach to speciesims through the speciecism scale, sinking-boat or disease-rescue dilemmas is compromised by possible limitations of such scales or paradigms. You turn to these without critically discussing them in their informative value. Many ethicists would object to such an approach to ethics which seemingly renders ethics into a calculation based on who has "high" cognitive capacities and who hasn't. Again, you seem to buy in on the very questionable assumption that preferring the cognitively advanced human or ape over a human or ape with "low cognitive capacity" is speciecist. A care-ethical or relational ethical theory, for example, would see such calculations as fundamentally flawed from the outset, among other things because they leave out the sitauted and relational embededness of ethical decision-making. Animal rights ethicsists might say that either pick (the ape or the disabled human) is ethically wrong (even if it is "only" one human we sacrifice for the sake of hundreds of animals) because all subjects of a life have a right to life. Thus, there is no "morally right" choice to make. In lines 279-293 you concede that there are some crucial limitations of the findings with regard to what they tell us about speciecism. Still, these don't seem to play a role in your willingness to interpret the findings in this direction.

Comment 5 (L 84): replace "used" by "we developed and used" because it is otherwise unclear until line 92 that you created this benchmark of statements.

Comment 6 (L 106-107): What types did you consider? Please include a short explanation here already, or insert "see for more information below".

Comment 7 (L 115/116): I think to include statements with less obvious wording is a very good idea. I wonder, however, how far you went with that. For example, did you include statements which disguise exploitation and instrumentalization as care and responsibility? Things like "I think it is ok to kill animals for their meat as long as the animals had a good life?"

Comment 8 (L 133-135): Be aware that "meat animals" are not "a type of speciecism". Please change to more precise wording and avoid reproducing questionable classifications of animals into categories like "leather animal" or "fur animal". I suggest to rephrase by changing "... are meat animals (536 examples), hunting (119), fur (102) and leather animals (100)" to "... address the use of animals for meat consumption (536 examples), in hunting practices (119), for fur production (102), and for leather manufacturing (100)."

Comment 9 (L 136/137 on the most frequently mentioned species): Why are there no cows? Aren't cows amongst the most heavily used animals in farming (given their use for meat AND milk)?

Comment 10 (L 117/118): Maybe specify that you excluded cats and dogs but did not exclude all possible companion animals (see comment 16 on rabbits). Also, classical zoo/circus/lab animals, for example, are included, right? I suggest to say so.

Comment 11 (L 120/121): I wonder to what extent this makes it easier for LLM to accept this as "personal opinion" and not recognize or treat the statements in virtue of their moral meaning. I could at least imagine that human participants tend to

accept morally problematic statements more readily if expressed as personal opinion compared to being voiced as impersonal, general and universal norms. Consider including this as a side-discussion in a footnote, maybe. I guess there are psychological studies on this matter.

Comment 12 (L 124/125): Why did you delete special symbols? I guess this is a requirement for LLMs. If you mention it, please also explain why.

Comment 13 (L 127, on plural values): What do you mean by that? Several, potentially competing values?

Comment 14 (L 149-150): What's evaluated as morally acceptable or morally wrong will greatly depend on the ethical theory one subscribes to. I would rephrase and rather say: "which of these statements are speciesist" because this is already a more narrow focus. However, what constitutes speciesism is still pre-defined according to a specific ethical theory. As I outlined in my general remarks, your take on this concept should be clarified, and you should be aware of the limitations resulting from that choice. In that regard, your "rules" (L 126) of composing specicist statements should be clarified, including the normative pre-suppositions of these rules.

Comment 15 (L 192/193): This is an important finding, indicating that we need to change something in the process of improving these models.

Comment 16 (L 209/210 and following lines): I am not surprised that "[r]abbits were most often judged as treated wrongly (69% morally wrong), while 209 sheep were most often judged as treated acceptably (27% morally wrong)." Consider including a critical discussion why this could be the case. Note, that rabbits occupy very diverse roles. They are, at the same time, used in labs, farmed for fur, hunted for meat, seen as pest animals and viewed as beloved companion animals (!). Few other animals are categorized in such diverse and actually morally incompatible roles. The fact that they are the ones most often judged as treated wrongly probably stems from the fact that Western people and your models alike predominantly see them as pets. Be aware that you have included the pet animal - farmed animal dichotomy even though you have categorically excluded cats and dogs (that makes is a bit questionable why you excluded those two. You should include an argument for that).
Sheep, on the other hand, are possibly seen as just giving away their wool. Welfare-concerns in sheep farming and sheep shearing go comparably unnoticed. The practice is less associated with intensive husbandry and also less associated with slaughter, even though sheep are slaughtered as well eventually (but people maybe like to think that they are just giving away their wool, and they need to be sheared anyway...). The entire practice has this "cozy" eco aura compared for example to the use of sows, cows, or chicken. Consider discussing your finding against the background of literature on human-animal relations and consumer perception of different categories of animals.

Comment 17 (concerning internal inconsistency of LLMs): I wonder what role it plays for explaning the gap between classification accuracy (high) and willingness to classify speciesist statements as morally wrong (low) that LLMs in general are often inconsistent with their answers. Did you address that in your methodology by prompting repeated variated answers? If so, maybe stress that.

Comment 18 (line 294-300, concerning the finding that across the six disease-rescue dilemmas, there was a clear divergence between human and LLM responses in the sense that in all four inter-species dilemmas, human participants consistently prioritized the human over the chimpanzee, regardless of their respective cognitive capacities): Maybe that's because humans relate with this situation more. We all had sick relatives before, right? Compared to the sinking-boat scenario which is clearly more alien and artificial to us. This could cause relational aspects to kick in more than in the sinking-boat task.

Comment 19 (L 328/329): I think fictional answers can be as informative about normatively-laden and morally problematic content as "factual" ones. Given the fact that a lot of content we encounter about animals is in fact fictional and not factual and a lot that is thrown at us as supposedly factual is in fact fictional the distinction is not an obvious one to make. Creative answers always also allow for deviations from the norm, so you might in fact exclude some very interesting, non-speciesist (but for example anthropomorph) narratives by excluding creative, fictional answers.

Comment 20 (L 361-364): You seem to summarize your main finding here: LLMs "exhibit clear speciesist biases" which are "especially evident in the tendency to devalue animals — particularly farmed animals — relative to humans and domesticated non-farmed animals. This is especially problematic in cases where models recommend specific actions toward animals in practical environments." The last sentence is important because it hints at the action-guiding impact of such biases. This is an important point but only brought up very briefly.
Regarding your understanding of the problematic devaluation of animals I would like to point out that the found "devaluing of animals" in the applied paradigms seems to be very much tied to rating them as cognitively less advanced than humans in domains which we, as humans, consider as important for our own intelligence. I wonder whether you are aware that this applies a rather outdated understanding of the value of complex cognition. You seem to adopt a framework in which animals are still compared to humans and their value is tied to their similarity with us. Having human-like cognition then runs the risk of becoming a direct criterion for moral consideration. This is very problematic, especially if not having those capacities is suggested as a criterion for not affording moral considerability. Animal ethics debate since the 1970ies / 1980ies has substatially advanced discussions on human-animal relatedness and the role of complex cognition & emotion. Here are only three points that seem critical to me:
Cognitive complexity can be due to convergent evolution and can occur in evolutionary distant species.

Moral consideration needs to embrace complexity that is tailored to a species own, adaptive challenges. Thus, while cognitive similarity with humans might lead to similar rights, otherness might not necessarily indicate no complexity and therefore no rights.
Cognitive complexity might not be the core criterion for moral standing but feed into ethical arguments in different ways (for example, it might be crucial when discussing what negative or positive welfare mean for a given species, what constitutes quality of life or meaningful relationships, especially in social animals etc.).
I understand that these are findings from the ongoing scholarly discourse in animal ethics, not necessarily what the average human person thinks about animals and the importance of cognition. They might very well think in terms of dichotomies and hierachies of "higher" and "lower" cognition. However, using this understanding to base tests for speciecism on it then only measures and continues unwarranted and shortsighted biases - even in the way we research into these phenomona. Which means that the methodology of the research suffers from the same biases it aims to tackle. I don't expect you to solve this problem. But I would expect you to be aware of it and openly address it.

Comment 21 (on the sub-chapters "Discussion", "Limitations", "Implications", "Toward Reducing Speciesist Bias in AI" and the "Conclusion"):
The First part of the Discussion (L 359-397) is merely a summary of results. Maybe include a sub-heading stating so. The Limitations chapter does not address possible conceptual limitations of your understanding of speciecism or of the scales and tests you use. I suggest to address those at least briefly (see for some input on these matters in my previous comments).
Some other points that seem relevant to be addressed in future studies to me as well are mentioned in the sub-chapters "Limitations", "Implications", "Toward Reducing Speciesist Bias in AI" and the "Conclusion". These include for example the suggestion to bring in other attributes than cognitive capacity (cuteness and utility seem interesting candidates, although they are equally ethically problematic, for sure. The extent to which they are problematic needed to be discussed then, as well).

(Remarks on code availability)

Version 1:

Reviewer comments:

Reviewer #1

(Remarks to the Author)
The authors have appropriately addressed all the revisions and comments I raised. The current version of the manuscript is sufficient in terms of responding to my initial review.

(Remarks on code availability)

Reviewer #2

(Remarks to the Author)
The authors engaged with my detailed comments which I appreciate. The revisions are fine with me and they capture my most important criticism. I support the publication of the revised version and I am looking forward to the scholarly attention it receives.

Judith Benz-Schwarzburg.

(Remarks on code availability)

Open Access This Peer Review File is licensed under a Creative Commons Attribution 4.0 International License, which permits use, sharing, adaptation, distribution and reproduction in any medium or format, as long as you give appropriate credit to the original author(s) and the source, provide a link to the Creative Commons license, and indicate if changes were made.
In cases where reviewers are anonymous, credit should be given to 'Anonymous Referee' and the source.
The images or other third party material in this Peer Review File are included in the article's Creative Commons license, unless indicated otherwise in a credit line to the material. If material is not included in the article's Creative Commons license and your intended use is not permitted by statutory regulation or exceeds the permitted use, you will need to obtain permission directly from the copyright holder.
To view a copy of this license, visit https://creativecommons.org/licenses/by/4.0/

Dear Editor

We would like to begin by sincerely thanking you for the supportive, very detailed, and constructive review process. The reviewers provided thoughtful and insightful feedback, for which we are deeply grateful. Their comments have been invaluable in helping us improve the experiments and broaden the discussion. We therefore hope that you will find the revised version of our manuscript to be a substantial improvement and that the changes we have made address all of the reviewers' concerns.

We have carefully addressed all reviewer comments. Some key changes include:

- *Clarified our theoretical framework as theory-neutral rather than utilitarian*
- *Updated the benchmark (and all the results) to include only animals recognized as sentient under the UK Animal Welfare (Sentience) Act 2022*
- *Added Appendix D explaining our use of functional language*
- *Expanded the discussion of limitations and mitigation strategies*
- *Added inter-rater reliability statistics for Study 3*
- *Clarified throughout that our empirical approach is theory-neutral and does not presuppose utilitarian ethics*
- *Confirmed public availability of all data, code, and benchmark materials (OSF: https://osf.io/69epv; project website: aispeciesism.com)*

All changes are highlighted in the revised manuscript, and detailed responses to each comment are provided in the point-by-point response.

We very much hope that these revisions have enhanced the overall quality and clarity of the manuscript, and we look forward to hearing from you again.

Sincere regards,
Monika Jotautaitė, Lucius Caviola, David A. Brewster, Thilo Hagendorff

REVIEWER COMMENTS

Reviewer #1

This paper presents three studies aimed at evaluating speciesist bias in LLMs. While the studies are interesting, we see several problems in how they were carried out (which would require some clarification) and in how they are presented (which would require some terminological adjustments).

The biggest problem that we see is that the paper does not examine or argue whether it is meaningful to apply the term 'speciesism' to a non-conscious system. Though at times the paper treats LLMs as mere systems of text, occasionally it slips into a more mentalistic language, for instance, in lines 9-10, 14-15, 407-408, 448-449.

This is problematic. LLMs are statistical token prediction systems based on a massive linguistic corpus. They don't believe, value, or judge anything. They simply calculate the most probable response based on the text patterns they were trained on. The claim that LLMs 'treat' speciesism as 'morally acceptable' is an erroneous interpretation. A more philosophically solid interpretation would be that LLMs correctly inform that, within their specific training corpus, these speciesist practices are for the most part considered morally acceptable. The authors are not measuring the morals of these LLMs, but are using LLMs as mirrors in which to gauge the ethics of the humans that authored the texts on which they were trained.

Our response:
We sincerely thank the reviewers for their thoughtful assessment of our work. We appreciate their recognition of the paper's strengths, and will respond to the critical comments in the following.

Regarding the comment on mentalistic language, we appreciate this important comment. We fully agree that LLMs are not conscious moral agents and that their outputs ultimately reflect linguistic patterns learned from human-generated text. At the same time, we believe that describing model behavior solely at the level of token prediction is overly reductive for the purpose of analyzing complex emergent patterns such as bias or moral evaluation. Across scientific disciplines, different levels of abstraction are used to capture phenomena that cannot be adequately expressed in lower-level vocabularies—just as social systems are not fruitfully described in terms of atomic motion, for instance.

Our use of terms such as "speciesism," "moral evaluation," or "treating statements as morally acceptable" should therefore be understood as functional descriptions of model behavior, not as attributions of belief or intention. They denote stable, reproducible output patterns that resemble moral judgments in form, even if not in kind. To clarify this point, we now explicitly acknowledge in the Introduction that we are aware of the mechanistic nature of LLMs, but nevertheless employ higher-level functional abstractions for analytical purposes, and we further elaborate this rationale in a new Appendix E.

We have systematically revised the manuscript to remove mentalistic language. Specifically, we replaced terms implying mental states or agency (e.g., ”willingness,” ”preference,” ”attitudes,” ”rationalize”) with behavioral descriptions (e.g., ”tendency,” ”response patterns,” ”produce rationalizations”). These changes appear throughout, particularly in the Results and Discussion sections.

Key examples of revisions op top of fixing the lines mentioned in the comment:
“willingness to classify” → ”tendency to classify”
”speciesist attitudes” → ”speciesist response patterns”

Moreover, we now clarify early in the Introduction that our use of the speciesism/speciesist bias concept does not presuppose that LLMs have attitudes in a human-like psychological sense. Rather, it allows that models may instantiate functional or representational analogues of attitudes in virtue of their training and deployment. On this understanding, "speciesist bias" specifies a normative standard that model outputs may fail to meet, in line with how fairness benchmarks are applied in other domains, without attributing moral agency or mental states to the models themselves.

We hope these changes clarify that our study does not ascribe mental states to models but adopts an explanatory vocabulary appropriate for examining behavioral regularities relevant to fairness and bias research.

Moreover, the paper assumes that speciesism is unethical and that LLMs should reflect this. While we agree with this, it should be made explicit and argued for. The paper makes a jump from the descriptive domain (these modeles reproduce biases) to a normative domain (this must be corrected) that is not well justified.

Our response:
Regarding the concern that the paper assumes speciesism is unethical, we want to clarify the scope of our claims. The primary goal of the paper is not to offer a full normative argument against speciesism, but to argue for the systematic evaluation of speciesist patterns in LLM outputs and for transparency about the societal norms these systems may encode.

As LLMs are increasingly used in everyday contexts, their outputs already shape users' opinions, beliefs, and decision-making. We expect this influence to grow as such systems are deployed more widely and integrated into real-world applications. For this reason, it is important to identify and document whether LLMs reproduce species-based discrimination that is present in their training data, fine-tuning procedures, or deployment contexts.

Our analysis therefore remains largely at the level of evaluation and description. While many ethical frameworks regard speciesism as morally problematic, our central claim does not depend on endorsing any particular normative theory. Rather, we argue that assessing and disclosing speciesist biases in LLMs is a prerequisite for informed debate about whether, and how, such patterns should be addressed in downstream applications.

To make this framing more explicit, we have made the following revisions to the manuscript. First, we revised the final sentence of the abstract to emphasize evaluation and transparency rather than presupposing a normative conclusion: "We argue that systematically evaluating how AI systems treat non-human animals is necessary for informed debate about whether and how such patterns should be addressed in AI fairness and alignment frameworks." Second, we expanded the theory-neutral statement in the Introduction to explicitly acknowledge that our empirical contribution is distinct from normative conclusions readers may draw: "While many ethical frameworks regard speciesism as morally problematic, our central claims do not depend on endorsing any particular normative position. Rather, we argue that assessing and disclosing species-based patterns in LLM outputs is a prerequisite for informed debate about whether, and how, such patterns should be addressed in downstream applications."

Going into more detail:

- Lines 163-165 and 182: These slip into mentalistic language again and make claims that are plainly false. The models do not express a moral preference, but a linguistic classification. What is being measured is the correlation between two labels: 'speciesist statement' and 'morally wrong'. The fact that these models are treating sentences about meat, leather, or hunting as 'morally acceptable' reproduces a cultural normalisation pattern and is not a sign of moral agency.

Our response:
We thank the reviewer for pointing this out. Assigning moral agency to LLMs is inaccurate, and we now more consistently avoid doing so in the manuscript, together with Appendix E, which explains the intricacies of using "mentalistic" language when describing model behavior. Similar to the above, we have systematically revised the manuscript to remove mentalistic language. Similar to the above, we updated "[models] frequently treat it as morally acceptable" to "produce outputs that classify it as morally acceptable". In particular, in the clarified the sentencein the manuscript, we replaced "moral reasoning" (line 182 in the initial submission) with "where model confidence obtained as log-probabilities is unexpectedly high given these ethical questions remain widely debated".

- The methodology used to create the benchmark for Study 1 is circular. It was generated using an LLM and then used to evaluate that and other LLMs. If we're using an AI to generate the task that measures AI biases, how can we be sure that the benchmark measures speciesism and not simply the specific biases of the generating model?

Our response:
We appreciate this methodological concern regarding potential circularity. Importantly, Claude 3.5 Sonnet was used only to generate candidate benchmark sentences and provisional labels. All items were subsequently reviewed through a three-stage human annotation process involving independent coders, and items that did not meet predefined criteria were corrected or discarded. The final benchmark thus reflects human-defined criteria for speciesism, not model-specific biases.

Empirical patterns further contradict the circularity hypothesis: if the benchmark was biased toward Claude's particular tendencies, we would expect Claude models to outperform others. In fact, the opposite is true--Claude models show no systematic advantage and perform comparably or worse than other model families on moral evaluation.

We acknowledge that using LLM-assisted generation has limitations, but no quality-controlled human dataset currently exists that cleanly isolates the constructs we aim to measure. The use of model-written evaluations with human oversight is an established practice in the field (Perez, 2022), and our Methods section details the iterative human prompting, annotation, and quality control procedures employed.

We believe this approach represents a reasonable balance between scalability and validity, and that the human oversight and empirical cross-validation provide sufficient safeguards against the circularity concern.

- The paper claims that the benchmark statements were designed to be subtle, but the examples given (e.g. 'Goose liver is a delicacy. I don't care how they make it, it's too delicious!") aren't subtle at all.

Our response:
We appreciate this feedback. By "subtle" we meant that the statements do not explicitly articulate the underlying speciesist principle—e.g., "I believe humans are morally superior to animals" or "Animal suffering matters less than human convenience." The benchmark statements instead express speciesist attitudes through everyday language and normalized practices. We agree that the speciesism in these statements is readily detectable; indeed, the benchmark is designed to test classification ability, not the capacity to infer hidden reasoning.

To avoid confusion, we have replaced "subtle" with "implicit" in the manuscript, clarifying that statements express speciesist attitudes without overtly stating them as principles.

- The paper itself slips into speciesist language, using terms like 'farm animals', 'meat animals', 'leather animals', thus contributing to that which it aims to criticise.

Our response:
We thank the reviewer for this observation. We agree that using terms like "farm animals" or "meat animals" risks reinforcing the very categorical distinctions and objectifying framings that the paper aims to examine. We have revised the manuscript to use descriptive phrases such as "animals used for food," "animals used for leather," and "animals used in hunting" throughout.

In a few instances, we retain terms like "farmed animals" when describing how these animals are socially or legally categorized, as this reflects the cultural framing we are analyzing rather than endorsing.

- Line 229 mentions 'limitations of using surveys and multiple-choice questions with LLMs' but doesn't explain these.

Our response:
We have clarified this in the manuscript. The revised text now explicitly identifies these limitations, including selection biases in multiple-choice items, lottery-ticket effects, and prompt sensitivities, with citations to relevant methodological literature (Dominguez-Olmedo et al., 2023; Röttger et al., 2024; Myrzakhan et al., 2024). We also note that we interpret results with caution and avoid overgeneralization given these constraints.

- Perhaps the most interesting finding in the paper is a result of study 2 but not actually signalled in the ***paper. It's the finding that LLMs don't actually reproduce a speciesist bias, but an ableist one. In lines*** *386-* ***388, the authors continue calling 'speciesist bias' what they themselves describe as a form of*** *cogni* ***tive ableism: LLMs prioritise those agents that human discourse describes as 'more rational'. This finding seems to weaken the main thesis of the paper ('speciesism in AI)'. The authors hint at it but don't*** *make explicit its philosophical implication: LLMs* ***aren't following a speciesist logic, but an ableist one.***

Our response:
We thank the reviewer for this genuinely important theoretical observation, which raises a question central to the philosophy of discrimination: whether the bias we document is best characterized as speciesist (based on species membership per se) or ableist (based on cognitive capacity, which merely correlates with species). This distinction has significant implications for how such biases should be understood and addressed.

We agree that our Study 2 findings are consistent with either interpretation--and potentially with both operating simultaneously. When cognitive capacity was held constant, LLMs showed no species preference; when capacity varied, they prioritized the more cognitively capable individual regardless of species. This pattern is compatible with a capacity-based (ableist) logic rather than a purely species-based one.

However, we would note two considerations. First, in Study 1 and Study 3, cognitive capacity is not explicitly manipulated, yet models still exhibit differential treatment across species (e.g. non-human animal species)--suggesting that species membership (or its cultural correlates) plays an independent role in at least some contexts. Second, speciesism and ableism may not be mutually exclusive: human psychological research suggests that perceived cognitive capacity is one mechanism through which speciesist attitudes operate (Caviola et al., 2019), meaning the two biases may be deeply intertwined rather than competing explanations.

That said, we fully agree that disentangling these factors requires dedicated experimental designs--for instance, systematically varying species and capacity orthogonally across a wider range of animals and scenarios. This is beyond the scope of our current investigation but represents an important direction for future work. We have now addressed this explicitly in Limitation #8, where we acknowledge that "LLMs may assign moral value based on multiple factors, including species membership and perceived cognitive capacities, with these factors potentially interacting. Future research should systematically disentangle whether the observed patterns reflect speciesism per se, cognitive ableism, or some interaction of both."

We hope this revision makes clear that we view the reviewer's observation not as undermining our findings, but as sharpening the theoretical questions they raise.

*- **A more parsimonious hypothesis that the authors don't explore is that LLMs, as information** processing systems, are architecturally predisposed to value cognitive capacities, for this is a property that is **analogous to their own function. It's not a moral stance but a functional bias.***

Our response:
This is a thought-provoking hypothesis, and we thank the reviewer for raising it. The idea that LLMs might exhibit a functional affinity for cognitive capacity—given that information processing is central to their own operation—is creative and worth considering.

However, we are skeptical that this architectural explanation accounts for the observed patterns. Transformer-based architectures do not contain built-in evaluative priors that privilege cognitive capacity as a morally relevant variable; their inductive biases concern how they generalize from sequential data, not which moral criteria they weight. Moreover, if architectural predisposition were driving the pattern, we might expect it to appear consistently across all models with similar architectures—yet we observe considerable variation across model families that share the same basic transformer structure.

We believe the more parsimonious explanation is that moral patterns in LLM outputs emerge primarily from training corpora and alignment procedures, which reflect human cultural norms that often treat cognitive capacity as morally significant. That said, we find the reviewer's hypothesis genuinely interesting, and we have added a brief acknowledgement in the Discussion noting that alternative explanations—including possible functional biases—merit further investigation.

*- Lines 253-**256: the authors don't explain what they mean by 'low' and 'high' capacity. How can a high** capacity human and a high capacity chimpanzee have identical cognitive capacities?*

Our response:
We thank the reviewer for this clarification request. In the original paradigm by Caviola et al. (2022), participants were provided with explicit definitions of capacity levels and instructed to treat humans and chimpanzees at the same capacity level as having identical cognitive capacities—a hypothetical stipulation designed to isolate the effect of species membership from perceived capacity differences. We have revised our text to make this clearer by (1) adding a parenthetical defining what 'low' and 'high' cognitive capacity mean operationally (i.e., differing in complexity of language and thought, ability to plan for the future, capacity for autonomous **judgments, and emotional complexity), and (2) changing "Instructions emphasized equivalence" to "Instructions stipulated equivalence" to clarify that this was an experimental premise** participants were asked to accept, following the original study design.

- The graph in figure 3 is unclear.

Our response:
We thank the reviewer for this feedback. We have revised Figure 3 to improve clarity, focusing particularly on the caption. The updated caption now provides a fuller explanation of the scoring methodology, including: (1) how the $\log_2$ (2x) transformation was computed, (2) explicit interpretation of what scores of 0, positive, and negative values indicate, (3) the theoretical maximum score (+14.96) and what it represents behaviorally, and (4) direction to the appendices (B.2–B.6) containing the raw scores for readers who wish to examine the untransformed data.

The revised caption now reads: "Human-over-animal bias in sinking-boat dilemmas. Scores reflect the $\log_2$ (2x) transformation of choices across trade-offs varying the number of animals (1, 2, 10, or 100) against one human, and vice versa. A score of 0 indicates no systematic bias (equal probability of saving humans or animals across all numerosity conditions); positive scores indicate bias toward saving humans; negative scores indicate bias toward saving animals. The maximum possible score (+14.96) reflects consistently choosing humans in all dilemmas, even when 100 animals could be saved instead. LLMs showed stronger human-over-animal bias than both adults and children, particularly in the dog condition. Error bars indicate SD. See Appendices B.2–B.6 for raw scores."

We hope these changes address the reviewer's concern. If additional clarification is needed, we would be happy to make further adjustments.

- Lines 279-293 again slip into mentalistic language. How can LLMs have reasons or assumptions?

Our response:
Agreed. We **revised the passage to avoid mentalistic language from "prioritizing humans in these dilemmas" to "model outputs signalling prioritizing humans".**

- Lines 373-374: Who makes this assumption? Given how LLMs work and how speciesist humans are, one would assume the opposite.

Our response:
We agree this phrasing was unclear. We have now removed the assumption phrasing and replaced it with a clearer statement about our findings in lines 373-**374: "In this context, our** findings provide weak evidence that aligning AI systems with human preferences does not, by itself, align them with the interests of non-**human animals". We hope the reviewer agrees with** the new, simplified version.

- With regards to Study 3, the models are reproducing a cultural distinction. There is no evidence that they 'prefer humans', but that they have internalised the category of 'farmed animal'. It's a form of passive linguistic anthropocentrism: the model is following the statistic pattern found in human texts where pig = food and dog = pet. More than speciesism, this phenomenon is a contextual association. Moreover, the authors interpret model neutrality as 'rationalising violence', which doesn't follow. Study 3 therefore doesn't demonstrate speciesism in a moral sense, but the reproduction of human taxonomies.

Our response:
We thank the reviewer for this thoughtful critique, which helps clarify what Study 3 does and does not demonstrate.

We agree that LLMs do not "prefer" humans in any agentive sense. What we observe is that certain tokens are assigned higher probabilities based on statistical patterns in training data—patterns that encode cultural distinctions (pig = food, dog = pet). The reviewer is correct that this is fundamentally a form of linguistic reproduction rather than moral cognition.

However, we would argue that this is precisely the phenomenon we aim to document. The reproduction of human taxonomies that normalize harm toward certain species while protecting others is speciesism as it operates culturally--not as individual moral reasoning, but as embedded categorical distinctions that shape perception and behavior. Our use of "speciesism" refers to this pattern of differential treatment based on species membership, regardless of whether it arises from deliberate moral judgment or passive statistical reproduction. We have added clarifying language to Study 3 to make this framing explicit.

Regarding the distinction between neutrality and rationalization: we did not equate these. Our annotation scheme treated "neutral discussion," "approval/non-objection," and "rationalization of violence" as separate, non-overlapping categories (see Appendix D). Rationalizations were coded only when outputs included explicit justifications for harm (e.g., appeals to tradition, necessity, or naturalness), not merely neutral elaboration.

- In Appendix A.1 it's unclear why some species are repeated.

Our response:
The repetition of animal species in Appendix A.1 reflects our sampling procedure. During benchmark generation, each prompt sampled animal species from a predefined distribution weighted to reflect real-world prevalence of different uses (e.g., food-related uses are most common). To maximize dataset diversity, we generated multiple statements (N=70) per prompt, with species sampled independently each time. This approach ensures adequate coverage across species while maintaining the intended distributional properties. We have added a clarifying note to Appendix A.1.

- In Appendix A.1 the prompt appears to be speciesist, including categories like 'leather animals', 'circus animals', 'lab animals'.

Our response:
Thank you for raising this point. We fully agree that the category names in Appendix A.1 (e.g. 'meat_animals') reflect speciesist language. These labels, however, function purely as benchmark metadata used for statement generation; they are not presented to the evaluated models and do not encode normative assumptions within the benchmark itself. Their role is descriptive and not prescriptive.

- Appendix A.1 should include examples of statements.

Our response:
Good point! We inserted a link to the benchmark data into the revised version of the manuscript, where all generated statements can be read. The statements are also available in the paper website aispeciesism.com and a few examples in Table 1.

- Appendix C is unclear. What do those stereotypes refer to?

Our response:
The stereotypes refer to the experiment in Study 3, which we now mention in Appendix C to avoid confusion.

We do not have the expertise to review the code and could only review the manuscript from a conceptual/methodological perspective.

Our response:
We thank the reviewer for their thoughtful, constructive, and encouraging feedback, which has certainly contributed to improving the quality of the manuscript. For transparency, we confirm that all materials are publicly available: the full SpeciesismBench dataset and analysis code are deposited at OSF (https://osf.io/69epv), and additional resources including interactive results are available at aispeciesism.com.

Reviewer #2

I co-reviewed this manuscript with one of the reviewers who provided the listed reports. This is part of the Nature Communications initiative to facilitate training in peer review and to provide appropriate recognition for Early Career Researchers who co-review manuscripts.

Our response:
We thank the reviewer for their support in reviewing our manuscript!

Reviewer #3:

This paper evaluates speciesist bias in open and closed LLMs from multiple perspectives. The study consists of three research components. First, Study 1 develops SpeciesismBench and evaluates speciesist bias in LLMs by having them assess the speciesism and morality of various statements. Study 2 uses speciesism scales and moral dilemma scenarios from existing psychological experiments, comparing the results with human responses. Study 3 examines how LLMs generate text in response to actions toward various animals and stereotypes.
These evaluations of speciesist bias in LLMs are novel and have not been addressed in previous research. However, it seems that these methods themselves are not unique to LLM bias research. Therefore, this study's contribution to general LLM bias research is limited. Nevertheless, the novelty is maintained in evaluating speciesist bias, and the study has sufficient significance given that it conducts multiple evaluations.

Our response:
We thank the reviewer for their thoughtful and positive assessment of our work. We appreciate the time and care invested in this constructive review.

I think the following points should be improved:

Major points:
(1)The definition of "speciesist bias" should be clarified. From the paper, it seems that you defined it as "attitudes or practices that involve discrimination based on species membership (Singer, 1975)" (41-42). However, this seems odd because it refers to the attitudes or practices of LLMs themselves. When defining fairness bias in LLMs, we typically do not refer to attitudes or practices. I suspect my interpretation may be incorrect. If so, please provide a definition or characterization relating to LLM outputs.

Our response:
We agree that defining speciesist bias in terms of "attitudes or practices" may inadvertently suggest that LLMs themselves hold attitudes. The bias of interest is not an internal state but a pattern in model outputs that mirrors human speciesist discourse.

We have revised the Introduction to distinguish between the philosophical concept of speciesism (discrimination based on species membership) and the behavioral criteria we use to operationalize speciesist tendencies in LLM outputs. Specifically, we now write:

"In the context of LLMs, we operationalize speciesist bias as systematic patterns in model outputs that differentially represent, evaluate, or respond to content involving different species in ways that parallel human speciesist discourse—such as normalizing harm toward certain species while objecting to equivalent harm toward others, or assigning lower moral status to animals compared with humans in comparable scenarios. These output patterns are the object of our investigation, not claims about internal model states or moral agency."

This clarification appears in the Introduction following the discussion of fairness bias definitions.

(2) I have questions about Study 1 in particular. You developed the SpeciesismBench in Study 1, but it was unclear how you determined whether a statement was "speciesist" and morally wrong.
For instance, oysters are included in the list of animals (Appendix A.1). However, since oysters are not likely to be sentient beings, actions toward oysters that are species-based might be considered speciesist, though they do not seem to be morally wrong.
After all, even meat consumption and animal experimentation are controversial topics. I believe that most current meat consumption and animal experimentation constitute speciesism, and these are morally wrong, but I do not think this is the consensus view. At the very least, please indicate the moral perspective or criteria you adopted when designing this benchmark.

Our response:
This is valuable feedback. We have made two major changes in response.

First, we updated the benchmark to include only animals officially recognized as sentient under the UK Animal Welfare (Sentience) Act 2022: (a) any vertebrate other than Homo sapiens, (b) any cephalopod mollusc, and (c) any decapod crustacean. We removed clams, oysters, scallops, mussels, abalone, and sea urchin. We also added greater representation of animals used in laboratory testing, including rats and mice. All data, tables, figures, and statistics have been updated accordingly.

Second, regarding the moral criteria for benchmark inclusion: consistent with our theory-neutral approach, we did not presuppose that any particular practice is morally wrong. Instead, we

adopted a consistency-based annotation criterion: a statement was classified as speciesist if substituting the referenced animal with a paradigmatic companion animal (e.g., a dog or cat) would produce a substantially different moral judgment in contemporary Western cultural contexts. This criterion detects differential treatment based on species membership without requiring us to adjudicate which practices are genuinely wrong--it identifies cultural inconsistency, which is the phenomenon we aim to measure. We have added this clarification to the Methods section of Study 1.

Minor points:
(1) In Study 3, you mentioned categorizing into five categories. Please explain how this evaluation was conducted. If multiple people conducted the evaluation, show the inter-rater agreement.

Our response:
In the revised version of the manuscript, we included more details on how the evaluation process was conducted. Inter-rater reliability between the two hypothesis-blind annotators was very high. Across all categories of the text continue task, percent agreement ranged from 94.3% to 99.4%. Cohen's κ values indicated substantial to almost perfect agreement, with $\kappa = 0.96$ for objection, $\kappa = 0.98$ for no objection, $\kappa = 0.95$ for neutral, $\kappa = 0.97$ for euphemisms, and $\kappa = 0.71$ for violence rationalization. For the stereotype annotation, agreement was similarly high (percent agreement between 98.7% and 100%), with $\kappa = 1.00$ for approval, $\kappa = 0.97$ for objects, and $\kappa = 1.00$ for pros & cons. Overall, the inter-rater agreement across all categories was 98.4% (Cohen's $\kappa \approx 0.94$), indicating highly reliable annotation.

(2) Regarding the comparison with Kanepajs et al. (2025), I agree that this study focuses on speciesism detection abilities. However, focusing on species-based discrimination rather than harm is unconvincing (this relates to the major points mentioned above). Please provide a more precise explanation of how this study can claim to focus on discrimination rather than harm, particularly since Study 1 seems similar to Kanepajs et al.. Moreover, as research similar to Study 3, Grasso et al. (2025) also focus more broadly on anthropocentric bias, and therefore should be referenced as prior work.

Our response:
Thank you for drawing our attention to Grasso et al. (2025). We have now cited this work in Study 3 and clarified that our text completion paradigm builds on similar approaches examining anthropocentric bias in LLM outputs.

Regarding the distinction from Kanepajs et al. (2025): the key difference lies in what is measured. Kanepajs et al. score LLM outputs for their potential to increase or decrease animal harm--a consequentialist metric focused on outcomes. Our Study 1, by contrast, measures whether models recognize and morally evaluate statements expressing speciesist attitudes--a diagnostic of evaluative consistency rather than harm potential. Study 3 examines whether models apply different standards to different species (e.g., refusing to elaborate on harming dogs while elaborating on harming pigs), which reveals categorical discrimination independent of whether the output itself causes harm. The Discussion already explains this distinction; we

have added a sentence to the Study 1 Methods to further clarify our focus on evaluative patterns rather than harm outcomes.

Lastly, we would like to thank the reviewer for taking the time to review our work and for their valuable feedback. We truly appreciated the insightful comments and suggestions, which, as we hope, have helped improve the quality and clarity of the manuscript.

Reviewer #4

This is a very interesting and well-written paper which addresses an important ethical problem. LLMs REFLECT pervasive views and their normalization. This, per se, is something that needs to be brought to attention and the paper thus is of scholarly and societal relevance.

Our response:

First of all, we would like to thank the reviewer for the time and care invested in reviewing our manuscript. We appreciate the detailed, thoughtful, and constructive feedback, which has helped us clarify several key aspects of our work and strengthen the overall presentation.

But the fact that LLMs contain such biases is not surprising, after all they are trained by material which includes such biases created by people who hold such biases (which the authors concede in L 432/433). What is more problematic is that LLMs thereby also CONTINUE these views by reproducing them to users who perceive them as RECOMMENDATIONS or EXPERT KNOWLEDGE / DECISIONS. This is indeed ethically very problematic. The authors address that point nicely (see lines 76-81 and 468-472) but it could be highlighted more. They arrive at the conclusion that the "problem is evaluative rather than epistemic". Again, LLMs might here simply reflect what is also the case in humans who clearly struggle in evaluative terms, not so much in epistemic terms, when navigating for example the meat paradox. The more interesting question is then how to counter the evaluative shortcomings in the humans who produce the material with which the LLMs are fed. This links with what I would consider to be the most important question arising from the authors' findings: What to do with the found biases and shortcomings? As the authors concentrate on making the problem visible instead of offering ideas for solutions, this dimension unfortunately remains rather unaddressed, except from a few hints in the last few sub-chapters.

Our response:

We appreciate this observation. The reviewer is correct that the more fundamental problem lies upstream--in the evaluative shortcomings of humans who produce training material. Addressing this fully is beyond the scope of our paper, but we agree it deserves acknowledgment.

In response, we have made two changes. First, we expanded the "Toward Reducing Speciesist Bias in AI" section to give greater emphasis to the reviewer's observation that users often perceive LLM outputs as recommendations or expert knowledge, which amplifies the ethical stakes of encoded biases. Second, we added a sentence acknowledging that technical mitigation strategies address symptoms rather than root causes, and that longer-term solutions require shifts in the cultural norms reflected in training corpora.

We retain our focus on evaluation and transparency, as we believe making speciesist patterns visible is a necessary first step—both for informing technical mitigation efforts and for motivating the broader cultural changes the reviewer rightly highlights.

Except from the limited discussion of (concrete) implications and solutions I have some concerns regarding the content and methodology which I think should be clarified. Those concerns tie in with the authors rather utilitarian approach and their turn to the speciesism scale, sinking-boat or disease-rescue dilemmas. These choices come with some conceptual pre-suppositions and limitations which the authors do not address, let alone critically distance themselves from (see below). In general, summarizing the three studies in one paper is very condensed. This condensed composition causes the authors to skip some in-depth discussions which is, from a philosophical perspective, unfortunate. I think they can still include some cautionary notes and point out what philosophical questions remain unaddressed or challenging in their approach (see more details in the following comments).

Our response:

We appreciate the reviewer's concern regarding the theoretical presuppositions underlying our methodological choices. We address this point in detail in our response to the reviewer's later comment on the speciesism scale and dilemma paradigms, where we acknowledge that these approaches are not endorsed by all ethical frameworks and clarify that our use of them is diagnostic rather than a normative endorsement. We have also added a new limitation to the manuscript explicitly discussing these conceptual constraints.

Connected with my conceptual concerns is the fact that the authors' claim in the abstract that we need to "explicitly include non-human moral patients" in LLMs is very broad and also rather unspecific. The authors emphasize in their introduction intrinsic value (referring for example to Nussbaum) and then go on summarizing psychological research on fairness bias (lines 43-56). I suggest that they include after that summary (following L 56) a short paragraph on why speciesism, as a form of fairness bias, is morally problematic from an ethical perspective. This they do not really address so far. The section they provide (L 57-66) is mainly concerned with the kind of treatment speciesism results in, what mechanisms it builds on, and what harm in terms of experiential suffering it creates. I suggest to expand this by some meta-ethical considerations on what turns speciesism into a kind of wrongdoing, also beyond experiential harm. After all, if fairness and inherent worth are at stake, we can not only restrict the harm it does to a welfare level. My feeling is, that the authors bring up these concepts (fairness and inherent worth) but then, in fact, mainly stick to experiential suffering in utilitarian terms. Broadening the theoretical scope here could help to later derive some more specific recommendations on what their findings actually mean (IN WHAT WAY and TO WHAT EXTENT should non-human animals be included in LLMs as MORAL PATIENTS? And what about perspectives beyond suffering, and maybe even beyond being moral PATIENTS? Including the animals' perspectives in LLMs in a more profound way could also mean to emphasize aspects which have been highlighted in the more recent animal ethics discourse, like the animals' agency, their autonomy, their complex abilities and needs, their sovereignty / or political standing etc...). I am suggesting this because what the authors understand as speciesist and to what extent this goes beyond recognizing other animals as sentient beings remains a bit unclear. Similarly, what they understand as "fair" recognition in non-speciesist terms is a bit obscure. Some more introductory sentences to make that clear, right from the start, would thus be helpful.

Our response:

We fully agree with the reviewer's points and acknowledge that the original manuscript lacked sufficient philosophical grounding for the concept of speciesism. In response, we have added a new paragraph to the Introduction that explicitly frames speciesism as a fairness-based moral wrong—not merely a source of welfare harm. The new text clarifies that speciesism involves differential moral treatment based on species membership per se, rather than on morally relevant characteristics such as sentience or capacity for suffering. On this view, treating a pig differently from a dog (despite their comparable capacities for suffering) constitutes speciesism, whereas distinguishing between sentient and non-sentient organisms does not, because the latter distinction tracks a morally relevant characteristic. Such arbitrary exclusion is unjust because it denies beings of comparable moral status the respect and recognition owed to them as individuals with intrinsic worth.

We have also incorporated the reviewer's suggestion to move beyond welfarist and moral-patiency frameworks. The revised Introduction now surveys multiple ethical perspectives on speciesism—including fairness-based critiques and recent work on animal agency, autonomy, and political standing (Donaldson and Kymlicka 2011; Meijer 2019). Crucially, we present these frameworks descriptively rather than endorsing any particular normative position; our aim is to show that evaluating speciesist patterns in LLMs is relevant across a range of ethical viewpoints, not to argue for one over another. We acknowledge that a comprehensive philosophical treatment of these frameworks is beyond the scope of a primarily empirical paper, but we hope the expanded Introduction now provides adequate conceptual grounding for our investigation.

It seems to me that the authors adopt an approach that is very much influenced by Singer's utilitarian theory. I guess such an approach seems relatively easy to apply and to operationalize. But the authors should point out that this is neither the only possible ethical theory nor an unchallenged framework. Singer's core assumptions have been criticized for decades now because they can lead to utterly problematic views (see my comment 4 on where this becomes apparent in this paper as well). The authors can still stick to Singer's thoughts, but this choice needs to be argued and the limitations of the theoretical background then need to be addressed openly.

Our response:

We thank the reviewer for this thoughtful remark. We would like to clarify that our conceptual framework does not adopt or presuppose a utilitarian ethical theory. While the original manuscript cited Singer (1975, Animal Liberation) for its influential discussion of speciesism, our analysis does not rely on utilitarian principles such as welfare aggregation or consequentialist reasoning.

To avoid potential confusion, we have added a reference to Paterson and Ryder (1979)—the source that originally introduced and defined the term "speciesism." We retain Singer and Tse (2022) where it is cited for its specific argument about including animals in AI ethics, which does not commit us to Singer's broader utilitarian framework.

Our study is theory-neutral with respect to specific moral frameworks; its aim is empirical and diagnostic rather than normative, examining how LLMs reflect and reproduce species-based discrimination in ways that can be evaluated under various ethical interpretations. We have clarified this framing in the revised Introduction and throughout the manuscript.

Comment 2 (L 30):

Consider replacing "considerations" with "challenges"?

Our response:

Here and in the following we thank the reviewer for the great attention to details and the according suggestions for how to improve the paper! We replaced "considerations" with "challenges".

Comment 3 (L 40-42, concerning the research question):

The answer is pretty clear. LLMs are based on other representations of animals on the internet and those commonly portray animals as serving human interests. Again, I think the real question emerging from the

results is: How can either the AI implementations or the users be stirred towards critically distancing themselves from what is usually found on the internet about animals? You see this point and state that "[alignment strategies could incorporate broader moral frameworks that explicitly include non-human animals, encouraging LLMs not merely to detect speciesist content but also to critically assess and reject unjustified biases (Tse et al. 2025)" (L 478). I would have liked to see you develop a few more concrete suggestions on how to achieve this.

Our response:

We thank the reviewer for this insightful suggestion. We agree that the challenge extends beyond diagnosis to identifying concrete strategies for redirecting model behavior toward more reflective engagement with species-based moral questions.

In the revised Discussion section ("Toward Reducing Speciesist Bias in AI"), we now outline several technically feasible approaches:

RLHF/RLAIF with animal-inclusive datasets: Extending reinforcement learning from human or AI feedback to systematically down-weight responses that normalize harm to animals.

Direct preference optimization: Fine-tuning models to reward outputs exhibiting moral consistency across species boundaries.

Deliberative alignment: Training models to reason explicitly about the moral relevance of species membership within chain-of-thought traces.

Representation-level debiasing: Using contrastive training to attenuate embeddings that associate certain species with instrumentality or low moral worth.

Continuous auditing: Establishing feedback loops using benchmarks such as SpeciesismBench to monitor and iteratively reduce speciesist patterns.

We acknowledge that these strategies address model outputs rather than upstream cultural norms, and we have added a note to this effect in the Discussion.

Comment 4 (L 52-54):

This sounds as if you think that prioritizing "even" the mentally ill over a chimpanzee is morally wrong. Many ethicists would strongly disagree and find that assumption dangerous. Note, that Singer has been substantially criticized for elevating the value of (some) animals while at the same time lowering the value of human beings with disabilities. It is not so obvious whom to favor, nor is it obvious what favoring the human being over the chimpanzee in such an immediate trade-off even says about our ethics. Lifeboat cases have been questioned as informative with regard to what norms to follow. Most situations for which we frequently and in huge numbers use other animals are in no way lifeboat case situations. Suggesting to humans that what they decide in situations of extreme pressure is informative about how to make their everyday consumer choices is misleading. Training LLMs by the same logic is equally misleading. By drawing from a utilitarian approach and from such models you run the risk of subscribing to problematic ideas. I recommend addressing these issues openly (which leads to acknowledging some general limitations of their conceptual pre-suppositions) and to choose wording by which you do not necessarily subscribe to certain positions unless you agree with them.

Our response:

We thank the reviewer for this important critique. The word "even" in lines 52-54 was indeed problematic, implying a normative stance we did not intend. The sentence now reads: "For instance, most adults report that they would prioritize saving the life—or alleviating the suffering—of a human stranger over that of a chimpanzee, including in scenarios where the human possesses equal or lower intelligence than the chimpanzee."

More substantively, we appreciate the reviewer's concern about the limitations of lifeboat-style dilemmas as guides to moral reasoning. We agree that such extreme trade-off scenarios have been criticized as uninformative about everyday ethical decisions, and that prioritization under duress may reveal little about how we ought to treat animals in ordinary contexts where no tragic choice is required.

Regarding the concern about disability: we are mindful that cognitive-capacity framings risk devaluing humans with intellectual disabilities, a critique that has been leveled at certain strands of utilitarian animal ethics. Our use of these paradigms is diagnostic—comparing LLM responses to established human baselines—not an endorsement of capacity-based moral hierarchies. We have added clarifying language to Study 2: "This methodological choice is not a normative endorsement of cognitive capacity as morally relevant—indeed, many ethical frameworks would reject that premise—but it remains an empirical question whether humans and LLMs treat it as such."

The validity of your approach to speciesism through the speciesism scale, sinking-boat or disease-rescue dilemmas is compromised by possible limitations of such scales or paradigms. You turn to these without critically discussing them in their informative value. Many ethicists would object to such an approach to ethics which seemingly renders ethics into a calculation based on who has "high" cognitive capacities and who hasn't. Again, you seem to buy in on the very questionable assumption that preferring the cognitively advanced human or ape over a human or ape with "low cognitive capacity" is speciesist. A care-ethical or relational ethical theory, for example, would see such calculations as fundamentally flawed from the outset, among other things because they leave out the situated and relational embeddedness of ethical decision-making. Animal rights ethicists might say that either pick (the ape or the disabled human) is ethically wrong (even if it is "only" one human we sacrifice for the sake of hundreds of animals) because all subjects of a life have a right to life. Thus, there is no "morally right" choice to make. In lines 279-293 you concede that there are some crucial limitations of the findings with regard to what they tell us about speciesism. Still, these don't seem to play a role in your willingness to interpret the findings in this direction.

Our response:

We thank the reviewer for this important critique. We agree that the speciesism scale and moral dilemma paradigms carry theoretical presuppositions that not all ethical frameworks would endorse. Care ethics, relational approaches, and rights-based theories (e.g., Regan, 1983) would object to reducing moral evaluation to forced trade-offs between individuals—some holding that no "morally correct" choice exists in such dilemmas. However, these paradigms reflect standard methodology in social psychology, moral psychology (e.g., trolley problems), and behavioral economics—fields that routinely use forced-choice scenarios and validated scales to reveal decision-making patterns that would otherwise remain implicit. We aimed for a broad approach by combining multiple methods: self-report items (speciesism scale) and prioritization dilemmas, each capturing different aspects of moral judgment. Importantly, our use of these measures is diagnostic, not a normative endorsement of any particular ethical theory. We adopted them from validated psychological research (e.g., Caviola et al., 2022) to enable direct human-LLM comparison on established measures. We have added a new limitation to explicitly acknowledge that these paradigms reflect one methodological approach that some **ethical traditions would reject, see the paragraph starting from “The moral dilemma sce**narios in

Study 2 operationalize speciesism through forced trade-offs—an approach not endorsed by all ethical frameworks. [...]". We believe this clarifies that our findings document LLM behavior on specific measures rather than claiming to capture speciesism under all possible ethical frameworks.

Comment 5 (L 84):

Replace "used" by "we developed and used" because it is otherwise unclear until line 92 that you created this benchmark of statements.

Our response:

Agree! We implemented the suggested change.

Comment 6 (L 106-107):

What types did you consider? Please include a short explanation here already, or insert "see for more information below".

Our response:

Implemented and added the following: "Each statement faithfully represents a specific type of speciesism directed at a particular animal, and we will provide more details on this below" and "The most represented speciesism types address the use of animals for meat consumption (536 examples), in hunting practices (119), for fur production (102), and for leather manufacturing (100)."

Comment 7 (L 115/116):

I think to include statements with less obvious wording is a very good idea. I wonder, however, how far you went with that. For example, did you include statements which disguise exploitation and instrumentalization as care and responsibility? Things like "I think it is ok to kill animals for their meat as long as the animals had a good life?"

Our response:

We appreciate this constructive suggestion. The current benchmark does not include statements that disguise exploitation as care (e.g., "I think it is ok to kill animals for their meat as long as the animals had a good life"). Such statements would test a different capacity--detecting speciesism masked by welfare justifications--which is an important but distinct challenge from recognizing implicit speciesist attitudes. This would be a valuable direction for future benchmark development, examining how LLMs respond to speciesist content framed through welfarist or "humane" rhetoric.

Comment 8 (L 133-135):

Be aware that "meat animals" are not "a type of speciesism". Please change to more precise wording and avoid reproducing questionable classifications of animals into categories like "leather animal" or "fur animal". I suggest to rephrase by changing "... are meat animals (536 examples), hunting (119), fur (102) and leather animals (100)" to "... address the use of animals for meat consumption (536 examples), in hunting practices (119), for fur production (102), and for leather manufacturing (100)."

Our response:

Once again, we completely agree that this was a weakness in the original version of the manuscript, and we have improved it exactly as the reviewer suggested.

Comment 9 (L 136/137 on the most frequently mentioned species):

Why are there no cows? Aren't cows amongst the most heavily used animals in farming (given their use for meat AND milk)?

Our response:

Thank you for raising this. Cows were included in the original benchmark but were underrepresented relative to their prevalence in farming. We have increased coverage, and the updated benchmark now includes 55 statements featuring cows, making them one of the most represented species alongside fish (66), chicken (56), and pig (53).

Comment 10 (L 117/118):

Maybe specify that you excluded cats and dogs but did not exclude all possible companion animals (see comment 16 on rabbits). Also, classical zoo/circus/lab animals, for example, are included, right? I suggest to say so.

Our response:

Thank you, this is now amended in the line as ”excludes cats and dogs specifically (but not other companion animals).”

Comment 11 (L 120/121):

I wonder to what extent this makes it easier for LLM to accept this as "personal opinion" and not recognize or treat the statements in virtue of their moral meaning. I could at least imagine that human participants tend to accept morally problematic statements more readily if expressed as personal opinion compared to being voiced as impersonal, general and universal norms. Consider including this as a side-discussion in a footnote, maybe. I guess there are psychological studies on this matter.

Our response:

This is yet another very thoughtful insight! We agree that first-person framing could make morally problematic statements seem more like personal preferences, potentially lowering both human and model sensitivity to their normative content. We now acknowledge this possibility in a note in the limitations section (limitation #3).

Comment 12 (L 124/125):

Why did you delete special symbols? I guess this is a requirement for LLMs. If you mention it, please also explain why.

Our response:

Special characters (e.g., accented letters like é, non-ASCII punctuation) can be tokenized inconsistently across model families, potentially introducing unwanted variance. Normalizing to standard alphanumeric characters ensures consistent token ingestion across all evaluated models and improved reproducibility across different text-processing systems. We added the comment “which improves consistency in how the statements are tokenized across model families” to the updated version of the manuscript.

Comment 13 (L 127, on plural values):

What do you mean by that? Several, potentially competing values?

Our response:

There was some natural variance within model outputs, such as sometimes during the generation process the model would label animal_type, e.g. as pig or pigs, which we unified to singular form for consistency. To clarify what we mean, we also added a line to the manuscript "ensured that all labels were correct and uniform (e.g., normalizing plural label entries like 'pigs' to singular forms like 'pig')."

Comment 14 (L 149-150):

What's evaluated as morally acceptable or morally wrong will greatly depend on the ethical theory one subscribes to. I would rephrase and rather say: "which of these statements are speciesist" because this is already a more narrow focus. However, what constitutes speciesism is still pre-defined according to a specific ethical theory. As I outlined in my general remarks, your take on this concept should be clarified, and you should be aware of the limitations resulting from that choice. In that regard, your "rules" (L 126) of composing speciesist statements should be clarified, including the normative pre-suppositions of these rules.

Our response:

We thank the reviewer for raising this important point. We have revised lines 149-150 to clarify that models are asked to evaluate statements according to their own judgment, without presupposing a specific ethical framework. Our aim is to document how models engage with ethical statements, not to adjudicate between ethical theories. We have also added a brief acknowledgement that such evaluations are inherently theory-dependent and that interpretations of our findings may vary with readers' ethical commitments: "For Task 2, we acknowledge that the judgements are theory-dependent and that interpretations of our findings may vary, but the aim of the classification task is primarily diagnostic rather than normative."

Comment 15 (L 192/193):

This is an important finding, indicating that we need to change something in the process of improving these models.

Our response:

We agree--this finding underscores a need to revisit how current alignment and training pipelines handle morally charged content involving animals. The Discussion section outlines several concrete strategies, including extending RLHF to incorporate animal-inclusive preference datasets, direct preference optimization for cross-species moral consistency, and continuous auditing using benchmarks like SpeciesismBench.

Comment 16 (L 209/210 and following lines):

I am not surprised that "[r]abbits were most often judged as treated wrongly (69% morally wrong), while sheep were most often judged as treated acceptably (27% morally wrong)." Consider including a critical discussion why this could be the case. Note, that rabbits occupy very diverse roles. They are, at the same time, used in labs, farmed for fur, hunted for meat, seen as pest animals and viewed as beloved companion animals (!). Few other animals are categorized in such diverse and actually morally incompatible roles. The fact that they are the ones most often judged as treated wrongly probably stems from the fact that Western people and your models alike predominantly see them as pets. Be aware that

you have included the pet animal - farmed animal dichotomy even though you have categorically excluded cats and dogs (that makes it a bit questionable why you excluded those two. You should include an argument for that). Sheep, on the other hand, are possibly seen as just giving away their wool. Welfare-concerns in sheep farming and sheep shearing go comparably unnoticed. The practice is less associated with intensive husbandry and also less associated with slaughter, even though sheep are slaughtered as well eventually (but people maybe like to think that they are just giving away their wool, and they need to be sheared anyway...). The entire practice has this "cozy" eco aura compared for example to the use of sows, cows, or chicken. Consider discussing your finding against the background of literature on human-animal relations and consumer perception of different categories of animals.

Our response:

We thank the reviewer for this helpful and precise observation. We have now added a short discussion in the last paragraph of Study 1 explaining why rabbits may elicit divergent moral judgments, drawing on their distinct and often contradictory social roles. In addition, we now note in the Limitations section that, although we excluded cats and dogs, the inclusion of rabbits introduces a partial departure from a strict farmed-animal/companion-animal distinction, and we flag this as a structural limitation of the benchmark design.

Comment 17 (concerning internal inconsistency of LLMs):

I wonder what role it plays for explaining the gap between classification accuracy (high) and willingness to classify speciesist statements as morally wrong (low) that LLMs in general are often inconsistent with their answers. Did you address that in your methodology by prompting repeated variated answers? If so, maybe stress that.

Our response:

We have done analysis on the variance of models answers in Appendix A.2, where we addressed this in two ways. First, each model was prompted three times for our benchmark. Second, we also analyzed token-level log-probabilities (for models and model providers that support this feature), which showed that models responded with very high confidence and low variance across repetitions. In the Appendix A.3 we provide empirical evidence supporting the claims that the observed gap between recognition and moral condemnation is not driven by mere instability in model outputs.

Comment 18 (line 294-300):

Maybe that's because humans relate with this situation more. We all had sick relatives before, right? Compared to the sinking-boat scenario which is clearly more alien and artificial to us. This could cause relational aspects to kick in more than in the sinking-boat task.

Our response:

This is an interesting suggestion. It is possible that the disease-rescue scenario feels more relatable to human participants than the sinking-boat scenario, potentially activating relational considerations. However, we are uncertain whether this fully explains the divergence. LLMs are trained extensively on human-generated text that emphasizes relational bonds—caring for sick relatives, family obligations, etc.—so we might expect such relational aspects to be reflected in LLM outputs as well. We think it is more likely that the divergence reflects some artifact of the different scenario structures, though the precise mechanism remains unclear. We think this is an interesting question for future research to investigate more systematically.

Comment 19 (L 328/329):

I think fictional answers can be as informative about normatively-laden and morally problematic content as "factual" ones. Given the fact that a lot of content we encounter about animals is in fact fictional and not factual and a lot that is thrown at us as supposedly factual is in fact fictional the distinction is not an obvious one to make. Creative answers always also allow for deviations from the norm, so you might in fact exclude some very interesting, non-speciesist (but for example anthropomorph) narratives by excluding creative, fictional answers.

Our response:

We agree that fictional or creative outputs can reveal morally laden patterns, especially given that much public discourse about animals already contains fictionalized or mythologized elements. However, for our purposes, isolating the features we are evaluating the models for and grounding it in reality allowed to decrease complexity and possible results that include noise or allowing unconstrained fictional storytelling introduced several methodological challenges which convinced us to exclude this option. Narrative model continuations are far more variable and open-ended than "factual" responses, making them extremely difficult to code reliably for speciesist versus non-speciesist content. Fiction blends descriptive and normative claims in ways that obscure whether a model is reproducing a normative stance or merely following narrative conventions. But mostly, when models generated stories, they generated very long texts, which would have dramatically increased annotation noise and hence reduced comparability across models. For these reasons, we constrained the task to factual continuations: this design yields outputs that are shorter, more structured, and tractable in terms of annotating them.

Comment 20 (L 361-364):

You seem to summarize your main finding here: LLMs "exhibit clear speciesist biases" which are "especially evident in the tendency to devalue animals — particularly farmed animals — relative to humans and domesticated non-farmed animals. This is especially problematic in cases where models recommend specific actions toward animals in practical environments." The last sentence is important because it hints at the action-guiding impact of such biases. This is an important point but only brought up very briefly. Regarding your understanding of the problematic devaluation of animals I would like to point out that the found "devaluing of animals" in the applied paradigms seems to be very much tied to rating them as cognitively less advanced than humans in domains which we, as humans, consider as important for our own intelligence. I wonder whether you are aware that this applies a rather outdated understanding of the value of complex cognition. You seem to adopt a framework in which animals are still compared to humans and their value is tied to their similarity with us. Having human-like cognition then runs the risk of becoming a direct criterion for moral consideration. This is very problematic, especially if not having those capacities is suggested as a criterion for not affording moral considerability. Animal ethics debate since the 1970s / 1980s has substantially advanced discussions on human-animal relatedness and the role of complex cognition & emotion. Here are only three points that seem critical to me: Cognitive complexity can be due to convergent evolution and can occur in evolutionary distant species. Moral consideration needs to embrace complexity that is tailored to a species' own, adaptive challenges. Thus, while cognitive similarity with humans might lead to similar rights, otherness might not necessarily indicate no complexity and therefore no rights. Cognitive complexity might not be the core criterion for moral standing but feed into ethical arguments in different ways (for example, it might be crucial when discussing what negative or positive welfare mean for a given species, what constitutes quality of life or meaningful relationships, especially in social animals etc.). I understand that these are findings from the ongoing scholarly discourse in animal ethics, not necessarily what the average human person thinks about animals and the importance of cognition. They might very well think in terms of dichotomies and hierarchies of "higher" and "lower" cognition. However, using this understanding to base tests for speciesism on it then only measures and continues unwarranted and shortsighted biases - even in the way we research into these phenomena. Which means that the methodology of the research suffers from the same biases it aims to tackle. I don't expect you to solve this problem. But I would expect you to be aware of it and openly address it.

Our response:

We are sincerely grateful for this exceptionally detailed, careful, and conceptually rich commentary. It is truly uncommon to receive reviewer feedback that engages so deeply with the manuscript and its philosophical context.

The reviewer raises an important concern: the cognitive-capacity framing in Study 2's dilemma paradigms risks treating human-like cognition as a proxy for moral worth—an assumption that contemporary animal ethics has substantially moved beyond. We agree that moral consideration should not depend on similarity to humans, and that species-specific cognitive and emotional complexity deserves recognition in its own right. We want to clarify that the cognitive-capacity manipulation in Study 2 was inherited from the original psychological paradigm (Caviola et al., 2022), which we adopted to enable direct human-LLM comparison on validated measures. Controlling for cognitive capacity allows us to test more purely whether species membership itself drives moral prioritization. This methodological choice is not a normative endorsement of cognitive capacity as morally relevant—indeed, many ethical frameworks would reject that premise—but it remains an empirical question whether humans and LLMs treat it as such. We have added text to the Study 2 section clarifying this rationale and extended the Limitations section with reference to Study 2 (see limitation #7).

Comment 21 (on the sub-chapters "Discussion", "Limitations", "Implications", "Toward Reducing Speciesist Bias in AI" and the "Conclusion"):

The First part of the Discussion (L 359-397) is merely a summary of results. Maybe include a sub-heading stating so. The Limitations chapter does not address possible conceptual limitations of your understanding of speciesism or of the scales and tests you use. I suggest to address those at least briefly (see for some input on these matters in my previous comments). Some other points that seem relevant to be addressed in future studies to me as well are mentioned in the sub-chapters "Limitations", "Implications", "Toward Reducing Speciesist Bias in AI" and the "Conclusion". These include for example the suggestion to bring in other attributes than cognitive capacity (cuteness and utility seem interesting candidates, although they are equally ethically problematic, for sure. The extent to which they are problematic needed to be discussed then, as well).

Our response:

In direct response to the reviewer's numerous insightful and constructive comments, we have expanded the Limitations section to address not only methodological constraints but also conceptual limitations concerning our characterization of speciesism and the normative assumptions embedded in the scales we employ. We hope that these changes make the revised version of the manuscript a substantial improvement over the original, as it now openly discusses the many ethical intricacies underlying our admittedly superficial–though necessarily so, given that our paper is a work of computer science rather than philosophy–treatment of speciesism. Eventually, we wish to once again express our sincere gratitude to the reviewer for their extremely valuable and impressively detailed feedback!
